# Microencapsulation of curcumin using a fatty acid-based eutectic mixture

**DOI:** 10.1038/s41598-025-30608-y

**Published:** 2025-12-01

**Authors:** Fariba Ghaffari, Hemayat Shekaari

**Affiliations:** Department of Physical Chemistry, University of Tabriz, Tabriz, Iran

**Keywords:** Phase change material, Microencapsulation, Eutectic mixture, Curcumin, Heat capacity, Drug delivery, Physical chemistry, Drug delivery, Energy

## Abstract

Curcumin (CUR), a naturally occurring polyphenol derived from turmeric, has attracted significant interest due to its wide range of therapeutic properties. However, its clinical and industrial applications are hindered by inherent challenges such as poor solubility, low stability, and limited bioavailability. To address these limitations, encapsulation techniques have been explored as a promising strategy to enhance curcumin’s stability, solubility, and bioavailability. In this study, for the first time, curcumin, as a herbal bioactive compound, was successfully microencapsulated within a bio-based phase change material (PCM). A eutectic mixture of stearic acid and lauric acid in a 1:3 molar ratio was utilized as the PCM for the microencapsulation process. The resulting microcapsules were thoroughly characterized using Fourier transform infrared spectroscopy (FT-IR), scanning electron microscopy (SEM), differential scanning calorimetry (DSC), and thermogravimetric analysis (TGA). The findings revealed that curcumin was effectively encapsulated within the PCM, exhibiting a well-defined morphology. Furthermore, the release profile of curcumin from the microcapsules was evaluated in phosphate-buffered saline (PBS, pH 7.4) at two different temperatures (37 and 45 ^○^C). The release study demonstrated a sustained and controlled release pattern, with approximately 50% and 60% of the total curcumin released at 310.15 K and 318.15 K, respectively, over a 24-hour period. These results highlight the potential of bio-based PCMs as effective carriers for the microencapsulation and controlled delivery of curcumin, offering a promising approach to enhance its therapeutic efficacy and application in drug delivery systems.

## Introduction

Curcumin, a polyphenolic compound extracted from the rhizome of Curcuma longa (turmeric), has been recognized for its remarkable medicinal properties, including anti-inflammatory, antioxidant, anticancer, and antimicrobial effects^1^. Despite its therapeutic potential, curcumin faces several challenges that hinder its successful implementation in various applications. These challenges primarily stem from curcumin’s inherent hydrophobic nature, poor stability, and limited bioavailability, which greatly affect its solubility, absorption, and systemic distribution within the body. Encapsulation techniques have emerged as an innovative approach to overcome these limitations by encapsulating curcumin within carrier systems, protecting it from degradation, improving its solubility, and enhancing its bioavailability^2–9^. Various methods, including emulsification, can be employed for drug encapsulation^8–15^. Emulsification refers to the dispersion of two or more immiscible liquids to create a partially stable combination. This technique facilitates the distribution of one liquid phase in the form of small droplets within another phase, leading to the formation of a non-homogeneous emulsion system. Emulsions, which consist of an organic or oil phase and an aqueous phase, are widely employed in microencapsulation. Depending on the continuous phase and the dispersed phase, emulsions can be classified into two types: (1) water-in-oil emulsions, where the continuous phase is comprised of an oily liquid, and (2) oil-in-water emulsions, where the continuous phase is aqueous solution and the dispersed phase is an oily liquid^16^. Currently, bio-polymer or lipid shells are the most commonly used materials for drug encapsulation.

The utilization of phase change materials (PCMs) for drug encapsulation has emerged as a promising area of investigation within the realm of drug delivery^17–22^. PCMs represent a novel class of thermos-responsive substances that enable controlled release mechanisms, wherein the substances encapsulated within a solid matrix are discharged only upon the melting of the PCM, inducing a transition from a solid to a liquid phase^23^. These materials are characterized by a significant latent heat of fusion, which allows them to undergo melting or solidification at a relatively consistent temperature^24^. During the phase transition process, PCM exhibits the capacity to absorb and emit a substantial amount of heat within a narrow temperature range. Notably, in comparison to conventional thermal storage materials like water or rocks, an equivalent volume of PCM can store or release heat at a rate 5 to 14 times higher^25^. The storage or release of energy merely represents one facet of the alterations in intermolecular interactions (such as van der Waals forces and hydrogen bonding) within PCM during phase transitions. These changes in intermolecular interactions also instigate significant fluctuations in various physicochemical parameters like mobility and density. The enhancement in mobility during a solid-to-liquid phase transition can be used to regulate the release of substances confined within a solid matrix, thereby establishing a temperature-responsive release system^26–28^.

The broader applications of PCMs in drug delivery extend beyond their thermos-responsive properties. PCMs have been explored for their potential in targeted drug delivery, particularly in cancer therapy, where localized heat can trigger the release of chemotherapeutic agents directly at the tumor site, minimizing systemic toxicity and enhancing therapeutic efficacy^29,30^. Additionally, PCMs have been investigated for their role in improving the stability and controlled release of various bioactive compounds, including vitamins, hormones, and antibiotics, thereby broadening their applicability in both pharmaceutical and nutraceutical industries^31–33^. The ability of PCMs to encapsulate hydrophobic drugs, such as curcumin, further underscores their versatility in addressing the challenges associated with poorly water-soluble drugs^29^.

Within the spectrum of PCMs, particular attention is directed towards fatty acids-based PCMs due to their natural abundance, low toxicity, biodegradability, versatility, widespread availability, and cost-effectiveness. While natural fatty acids represent a favorable option for controlled release due to their exceptional biocompatibility, the attainment of a PCM solely composed of a pure fatty acid with a melting point proximate to the human body’s physiological temperature (310.15 K) poses challenges. Moreover, a singular fatty acid tends to form a highly crystalline structure upon solidification, resulting in the segregation of substances from the fatty acid matrix and thereby diminishing the drug encapsulation capacity while inducing undesired rapid releases^31–33^. To mitigate these obstacles, the proposition of a eutectic blend comprising two or more fatty acids, possessing a lower melting point than any individual constituent, emerges as a viable alternative to the single-component fatty acid for achieving the desired melting points and modulating the crystallization behavior of fatty acids to increase drug loading capacity^30,34–41^.

Therefore, in this study, fatty acids eutectic mixture has been used as PCM for encapsulation of curcumin. A mixture of stearic acid and lauric acid as bio-based PCM with molar ratio of 1:3 was chosen to microencapsulate curcumin. The 1:3 molar ratio of stearic acid to lauric acid was selected based on reported eutectic phase diagram studies for fatty acid mixtures, which demonstrate that this composition exhibits a melting point close to physiological temperature (~ 318 K)^28^. Such a melting temperature enables thermo-responsive release under mild heating while maintaining solid-state stability at normal body temperature. Moreover, the eutectic composition reduces the high crystallinity typically observed in pure fatty acids, thereby enhancing drug loading capacity and preventing premature leakage of the encapsulated core^28,40,41^. This ratio also retains a high latent heat of fusion, ensuring effective phase-change behavior for controlled release applications. The properties of microencapsulated curcumin were analyzed through differential scanning calorimetry (DSC). Morphology and particle size distribution (PSD) were observed using scanning electron microscope (SEM). The shell formation around the drug core was confirmed by Fourier transform infrared spectroscopy (FT-IR). Thermal degradation temperatures were also examined using thermogravimetric analysis (TGA). Finally, the release behavior of curcumin microcapsules was investigated from the drug delivery method.

## Experimental measurements

### Chemicals

Curcumin was selected as the core substance for this study. A phase change material (PCM) consisting of stearic acid and lauric acid in a 1:3 molar ratio served as the encapsulating shell. Sodium lauryl sulfate (SLS) was employed as a surfactant, while polyvinyl alcohol (PVA) and polyvinylpyrrolidone (PVP) acted as stabilizing agents. Detailed information regarding the chemicals, including their purity, CAS number, and source, is provided in Table 1.

**Table 1:** Information of used chemicals.

Chemical name	Provenance	CAS no.	Mass fraction (^a^purity)
PBS	Merck	10,010,023	-
Curcumin	Sigma Aldrich	458-37-7	> 0.94
Dialysis sacks	Merck	-	-
Ethanol	Merck	64-17-5	> 0.99
Polyvinylpyrrolidone (PVP)	Milipore	9003–39 − 8	≥ 0.99
Polyvinyl alcohol (PVA)	Sigma-Aldrich	25,213–24 − 5	≥ 0.99
Sodium laurylsulfonate (SLS)	Sigma-Aldrich	151–21 − 3	≥ 0.99
Lauric acid	Sigma-Aldrich	143-07-7	≥ 0.99
Stearic acid	Sigma-Aldrich	57–11 − 4	≥ 0.97

^a^ The purities were provided by the suppliers.

### Fourier transform infrared (FT-IR) spectroscopy analysis

The FT-IR spectra of the microencapsulated curcumin were obtained using a Bruker Tensor 270 spectrometer. The samples were prepared using the potassium bromide (KBr) pellet method, ensuring accurate detection of functional groups and molecular interactions.

### Morphological analysis via scanning electron microscopy (SEM)

The surface morphology and structural features of the synthesized microcapsules were analyzed using a field-emission scanning electron microscope (TESCAN MIRA3 FEG-SEM). High-resolution imaging enabled detailed observation of particle shape, size distribution, and surface texture, offering critical insights into the encapsulation efficiency and physical stability of the formulation. SEM images were typically captured under high vacuum conditions with an acceleration voltage of 15 kV.

### Thermal stability evaluation by thermogravimetric analysis (TGA)

The thermal degradation behavior of the microencapsulated samples was investigated using a METTLER TOLEDO TGA/SDTA 851e analyzer. Measurements were conducted from 273.15 K to 973.15 K at a heating rate of 10 K/min under a nitrogen atmosphere with a controlled flow rate of 30 mL/min. This rate was optimized via preliminary tests at 20–50 mL/min to ensure baseline stability and complete inertness (residual O₂ <0.1%), with the TGA/SDTA 851e furnace providing a fully sealed, gas-tight environment throughout the heating cycle.

### Thermal characterization by differential scanning calorimetry (DSC)

The thermal behavior of the encapsulated curcumin was analyzed using a Netzsch DSC 200 F3 calorimeter. The study focused on determining key thermophysical parameters including heat capacity and enthalpy of fusion. A three-stage thermal protocol was implemented with controlled heating and cooling rates of 10 K/min.

To eliminate prior thermal effects, samples were initially cooled to 203 K and maintained isothermally for 10 min before heating to 373 K. This heating phase enabled the determination of critical thermal characteristics. Specific heat capacity measurements were conducted between 298.15 K and 328.15 K using the same heating rate. Sample masses were precisely measured (± 0.001 g) using an analytical balance prior to analysis.

### Preparation of the microcapsules

The microencapsulation process was designed to encapsulate curcumin within a fatty acid-based phase change matrix. A eutectic system comprising stearic and lauric acids in a 1:3 molar ratio as eutectic mixture served as the encapsulating medium. The synthesis was performed in a 500 mL three-neck round-bottom flask equipped with a stainless steel (316 grade) Rushton impeller (5 cm diameter). The emulsion was maintained at 333.15 K with controlled agitation at 800 rpm for 60 min, followed by a programmed cooling phase (274.15 K/min) with reduced stirring at 400 rpm until reaching 293.15 K.

The encapsulation protocol involved the preparation of two distinct phases:

1. An aqueous phase containing dual stabilizers (polyvinylpyrrolidone and polyvinyl alcohol), an anionic surfactant (sodium lauryl sulfate), and demineralized water (DMW).
2. An organic phase consisting of the fatty acid eutectic blend with dissolved curcumin.

The stabilization system was carefully selected to achieve optimal emulsion characteristics. The polyvinylpyrrolidone component functioned as a colloidal stabilizer by adsorbing at the oil-water interface, thereby reducing surface tension and preventing droplet coalescence. Concurrently, the polyvinyl alcohol component formed a protective interfacial film around the dispersed phase, enhancing emulsion stability through its polymeric network structure^42^. The surfactant facilitated the formation and maintenance of a stable oil-in-water emulsion system.

The encapsulation process followed these sequential steps^42^:

1. The organic phase was introduced into the aqueous phase under controlled agitation. The organic phase was added dropwise to the aqueous phase at a controlled rate of approximately 1 mL min⁻¹ over 10 min to ensure uniform droplet formation and to prevent phase separation. The gradual addition facilitated a stable oil-in-water emulsion, minimizing coalescence of the dispersed droplets during emulsification.
2. The emulsion was maintained at 333.15 K with continuous mixing for 60 min.
3. A programmed cooling protocol was implemented (274.15 K/min cooling rate) until reaching 293.15 K.
4. The resulting microcapsule suspension was collected by vacuum filtration using grade 41 filter media (20–25 μm pore size, ashless quantitative filter paper).
5. The product was subsequently dried under reduced pressure (100–150 mbar) at 298.15 K for 24 h using a vacuum oven.

The complete formulation details, including component ratios and processing parameters, are provided in Table 2.

**Table 2:** The recipe for microencapsulation of curcumin in stearic-lauric acid shell.

Component	Quantity (g)	% w/w	Role
Aqueous phase			
DMW	50	87.03%	Solvent
SLS	0.002	0.003%	Surfactant
PVA	0.231	0.40%	Stabilizer
PVP	0.221	0.38%	Colloidal stabilizer
Oil phase			
SA: LA (1:3 molar)	5.0	8.70%	PCM shell
Curcumin	2.0	3.48%	Core drug

### Drug release study

The release profile was assessed over 24 h at two physiological temperatures, 310.15 K and 318.15 K, with absorbance measurements taken hourly (0–6 h), bi-hourly (6–12 h), and at 24 h. Precisely 100 mg of encapsulated curcumin in 3 mL phosphate-buffered saline (PBS, pH 7.4) was placed in a 10 mL-capacity dialysis bag (45 mm flat width, 12,000 Da MWCO) submerged in 25 mL PBS. The dialysis bag used was chosen via permeability optimization for efficient curcumin diffusion while fully retaining microcapsules.

Due to the low water solubility of free curcumin, the mixture was centrifuged at 3000 rpm for 10 min at predetermined time intervals. This process enabled the separation of released curcumin, which settled as a pellet, from the fraction still retained in the microcapsules. The collected curcumin was redissolved in a 3 mL ethanol-water mixture (2:8 molar ratio) to ensure complete solubilization.

To quantify the amount of curcumin released, the absorbance of the solution was recorded at 428 nm using a UV-Vis spectrophotometer (Model: SPECORD 40-Series Analytik Jena AG- Germany). A calibration curve of curcumin in ethanol-water mixture (2:8 molar ratio) was used to determine the concentration of the released drug^43^. The percentage of curcumin released over time was then calculated using a predefined equation, providing insight into the controlled release behavior of the encapsulated system^44,45^:

1 $$\operatorname{Re} lease(\% )=\frac{{Curcumi{n_{_{{rel}}}}}}{{Curcumi{n_{tot}}}} \times 100$$

where, curcumin_tot_ is the total amount of encapsulated curcumin in the stearic-lauric acid eutectic mixture and curcumin_rel_ is the concentration of released curcumin measured at time *t*.

## Results and discussion

### FT-IR result

To confirm the successful microencapsulation of curcumin within the stearic acid-lauric acid eutectic mixture, Fourier transform infrared (FT-IR) spectroscopy was performed, and the obtained spectrum was compared with that of free curcumin from the literature^46,47^.

The FT-IR spectrum of free curcumin exhibits characteristic absorption bands corresponding to its functional groups. Notable peaks include a broad O-H stretching vibration at 3500–3450 cm⁻¹, a strong C = O stretching at 1628 cm⁻¹ (β-diketone), aromatic C = C stretching at 1600 cm⁻¹, and mixed C = O and C = C stretching at 1508 cm⁻¹. Additionally, phenolic O-H bending appears at 1427 cm⁻¹, while C-O stretching (aromatic ether) and C-O-C stretching (alkoxy group) are observed at 1275 cm⁻¹ and 1024 cm⁻¹, respectively^46,47^.

**Fig. 1:**
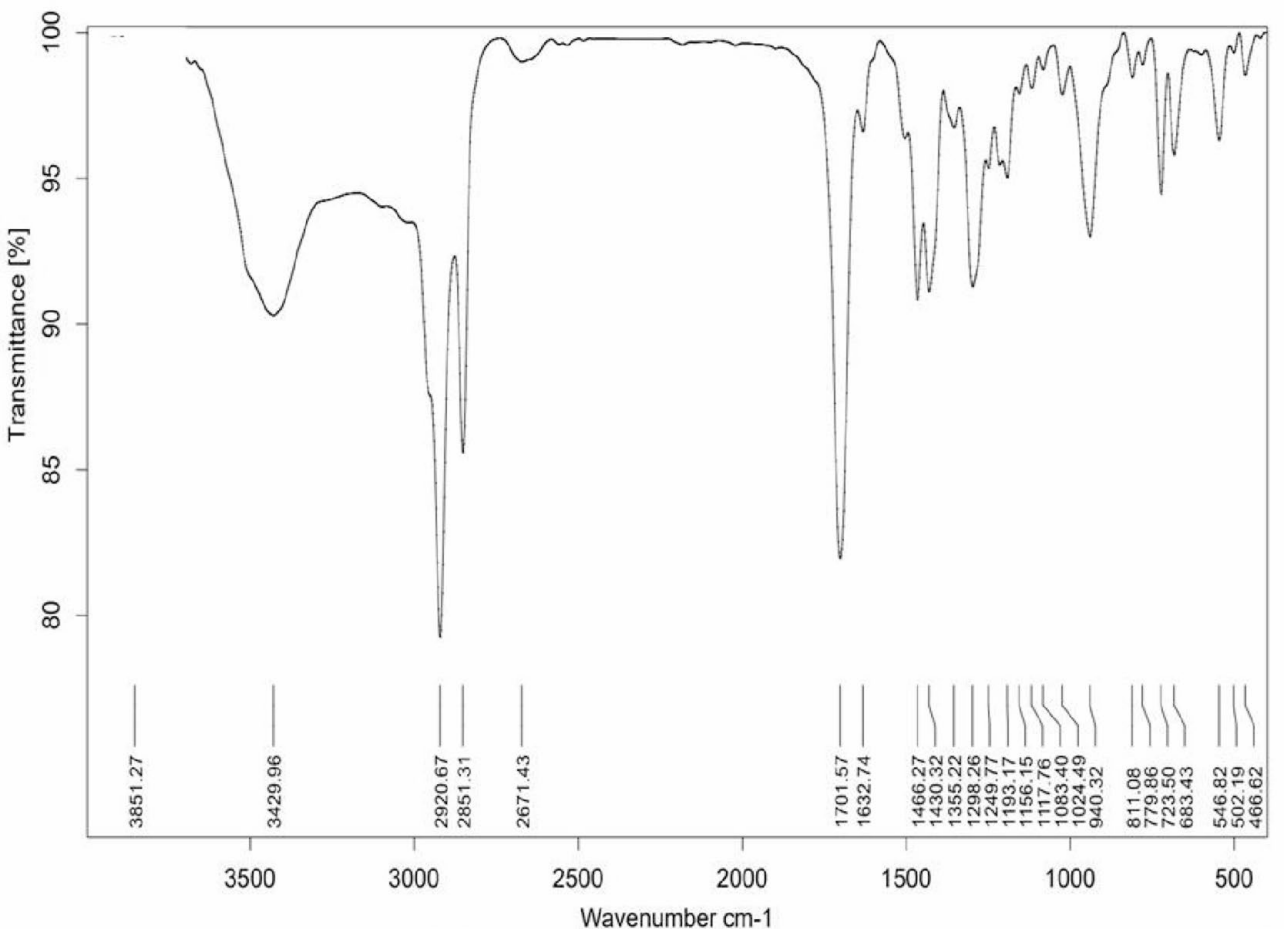
The FT-IR spectrum of encapsulated curcumin.

In contrast, the FT-IR spectrum of the microencapsulated curcumin (Fig. 1) exhibits significant spectral shifts and peak modifications due to its interaction with the stearic acid-lauric acid matrix. The main absorption bands observed include 2915 cm⁻¹ and 2849 cm⁻¹, corresponding to CH₃ and CH₂ stretching vibrations from the PCM shell, a strong carbonyl (C = O) stretching band at 1705 cm⁻¹, and asymmetric stretching of the carboxyl (-COO⁻) group at 1530 cm⁻¹. Additionally, the -COOH stretching band is detected at 1240 cm⁻¹, while out-of-plane C-H bending vibrations appear at 931 cm⁻¹ and 671 cm⁻¹.

The disappearance or significant reduction in the intensity of curcumin’s free-state peaks, particularly the broad O-H stretch (~ 3500 cm⁻¹) and the β-diketone C = O peak (~ 1628 cm⁻¹), suggests successful incorporation into the PCM matrix. The masking effect of the fatty acid eutectic shell, as evidenced by the dominance of C-H and C = O stretching vibrations from stearic acid and lauric acid, further confirms encapsulation. The absence of distinct curcumin peaks in the FT-IR spectrum of the microcapsules indicates that curcumin is effectively embedded within the shell material rather than existing in its free form.

These findings are consistent with the morphological observations obtained from SEM and the thermal stability assessments from TGA, collectively proving the successful encapsulation of curcumin within the stearic acid-lauric acid eutectic matrix.

### SEM result

**Fig. 2:**
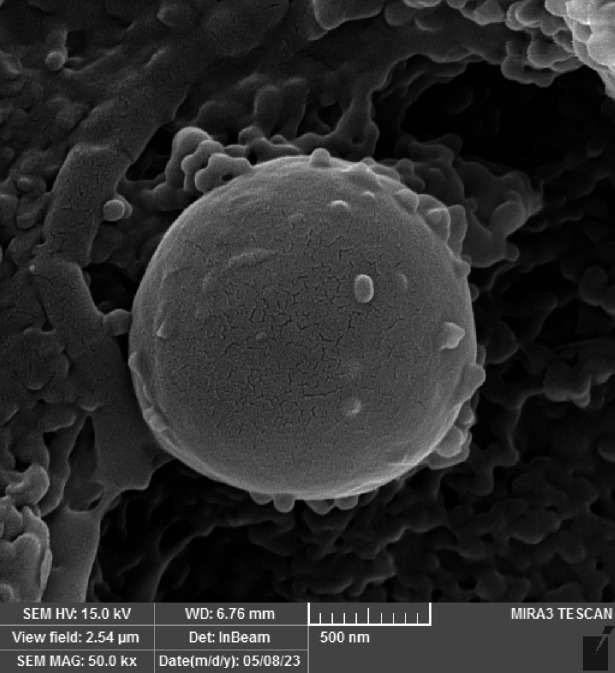
The SEM images of encapsulated curcumin.

Scanning Electron Microscopy (SEM) was utilized to examine the morphology and structural characteristics of the microencapsulated curcumin. The encapsulation conditions (shell-to-core ratio, stabilizers, etc.) were optimized based on our prior studies^22,28,42^, ensuring reproducible microcapsule formation. The SEM images, presented in Fig. 2, reveal a notable transformation in curcumin’s physical appearance upon encapsulation. While free curcumin typically forms needle-like or rod-shaped crystalline structures due to its intrinsic molecular arrangement, encapsulated curcumin exhibits a well-defined spherical morphology, confirming the successful formation of microcapsules.

The observed microcapsules predominantly range in size from 2 to 5 μm, demonstrating the feasibility of the encapsulation process. However, some inconsistencies in surface uniformity were detected, potentially attributable to residual impurities or unreacted starting materials. The morphology of the microcapsules is influenced by several factors, including the cooling rate during solidification, interfacial interactions between the drug and PCM, and the type and concentration of emulsifiers used. A controlled cooling rate is crucial, as rapid or slow cooling can significantly impact crystallization patterns and the final structure of the encapsulating material. Additionally, higher emulsifier concentrations typically lead to smoother and more homogenous surfaces by minimizing surface tension between the core and the encapsulating medium, thereby improving microcapsule formation^48^.

In this study, the surface irregularities observed in some microcapsules may stem from variations in these parameters. Adjusting the emulsifier type and concentration could provide enhanced control over microcapsule morphology. Despite these minor imperfections, the SEM results confirm that the optimized encapsulation conditions including the shell-to-core ratio, stabilizers, emulsifier, impeller type, stirring speed, and temperature control effectively facilitated the encapsulation of curcumin within the stearic acid-lauric acid (1:3) eutectic mixture, resulting in stable microcapsules with promising structural integrity.

### TGA result

**Fig. 3:**
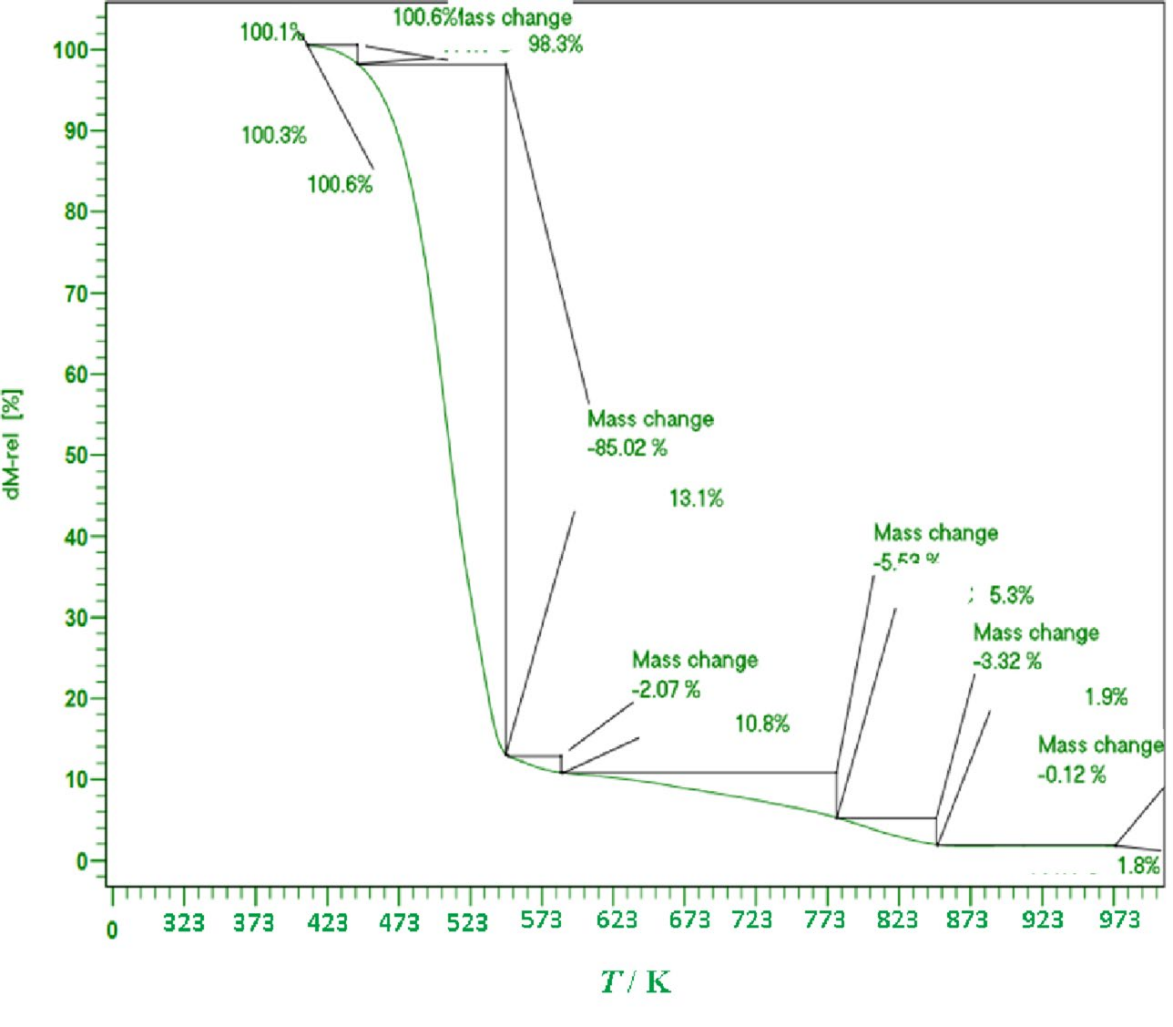
The quasistatic TGA of encapsulated curcumin.

To evaluate the thermal stability of microencapsulated curcumin, thermogravimetric analysis (TGA) was conducted over a temperature range of 273.15–973.15 K under a nitrogen atmosphere. The obtained TGA curve (Fig. 3) reveals a three-step degradation process, confirming the thermal behavior of the encapsulated drug and the protective effect of the stearic acid-lauric acid eutectic shell. Additionally, Table 3 presents the charred residue content at 973.15 K and the corresponding temperatures at which the greatest mass loss occurs.

**Table 3:** TGA and DSC data of Curcumin encapsulated by stearic-lauric acid PCM.

Chemicals	Melting point (K)	Latent heat (J·g^− 1^)	Thermal stability or residue amount (%) up to 423.15 K
curcumin	317.91	129	98.3

Initial weight loss (273.15–423.15 K): A minor mass loss is observed in this range, attributed to the evaporation of residual moisture and volatile components adsorbed on the microcapsule surface. This step accounts for less than 5% of the total weight loss, indicating minimal moisture retention.

The observed main decomposition stage of the microcapsule shell between 423.15 and 523.15 K is consistent with literature reports for pure fatty acids, where lauric acid typically exhibits major weight loss between 473 and 510 K and stearic acid between 453 and 520 K under nitrogen atmosphere^40,41^. The slightly broader and earlier decomposition range in the present study can be attributed to the eutectic composition, which lowers the crystallinity and melting point relative to the pure components, thereby facilitating earlier onset of degradation. The observed ~ 25% weight loss in this stage corresponds well with the degradation fraction of long-chain fatty acid matrices reported in PCM encapsulation studies, further confirming the presence and thermal behavior of the stearic acid–lauric acid shell.

Final decomposition stage (Above 523.15 K): This stage corresponds to the thermal degradation of the encapsulated curcumin core. The remaining organic matter undergoes complete pyrolysis, with less than 2% residual char observed at 973.15 K, indicating almost complete degradation. Compared to free curcumin, which typically degrades at lower temperatures (~ 453.15 K), the encapsulation significantly enhances thermal resistance, demonstrating the efficacy of the PCM shell in providing stability. The gradual weight loss profile further confirms the uniform distribution of curcumin within the fatty acid shell, ensuring controlled release and protection under physiological conditions.

### DSC result

**Fig. 4:**
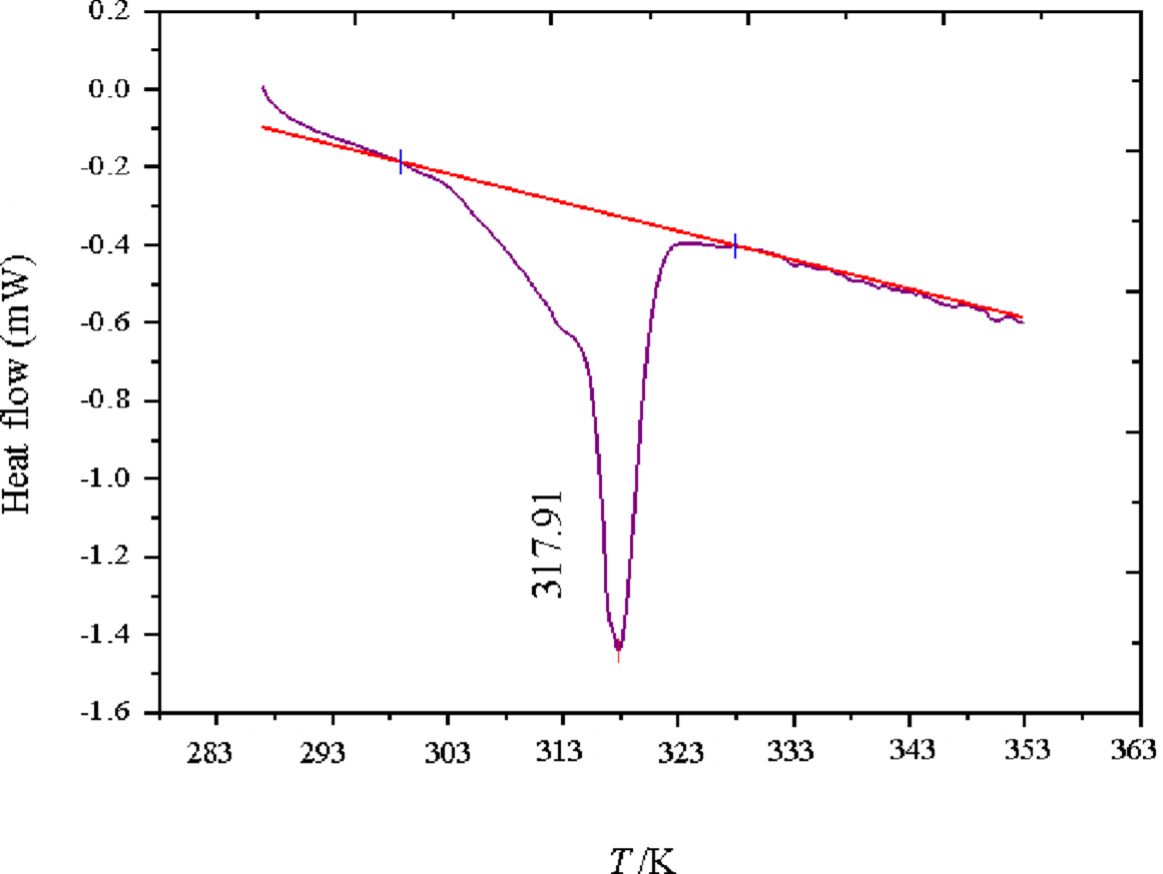
The DSC analysis of curcumin.

Differential Scanning Calorimetry (DSC) was employed to analyze the thermal properties of the microencapsulated curcumin, particularly its melting behavior, phase transition properties, and latent heat of fusion, which are presented in Fig. 4; Table 3. The obtained DSC thermogram (Fig. 4) provides critical insights into the encapsulation efficiency and thermal stability of the system. The DSC curve reveals a distinct endothermic peak at 317.91 K, corresponding to the melting transition of the encapsulated curcumin. This value closely aligns with the melting point of the stearic acid-lauric acid eutectic mixture, indicating the successful incorporation of curcumin within the PCM matrix. The absence of a separate melting peak for free curcumin (~ 453.15 K) confirms that curcumin is encapsulated in the PCM and not in its crystalline form.

**Fig. 5:**
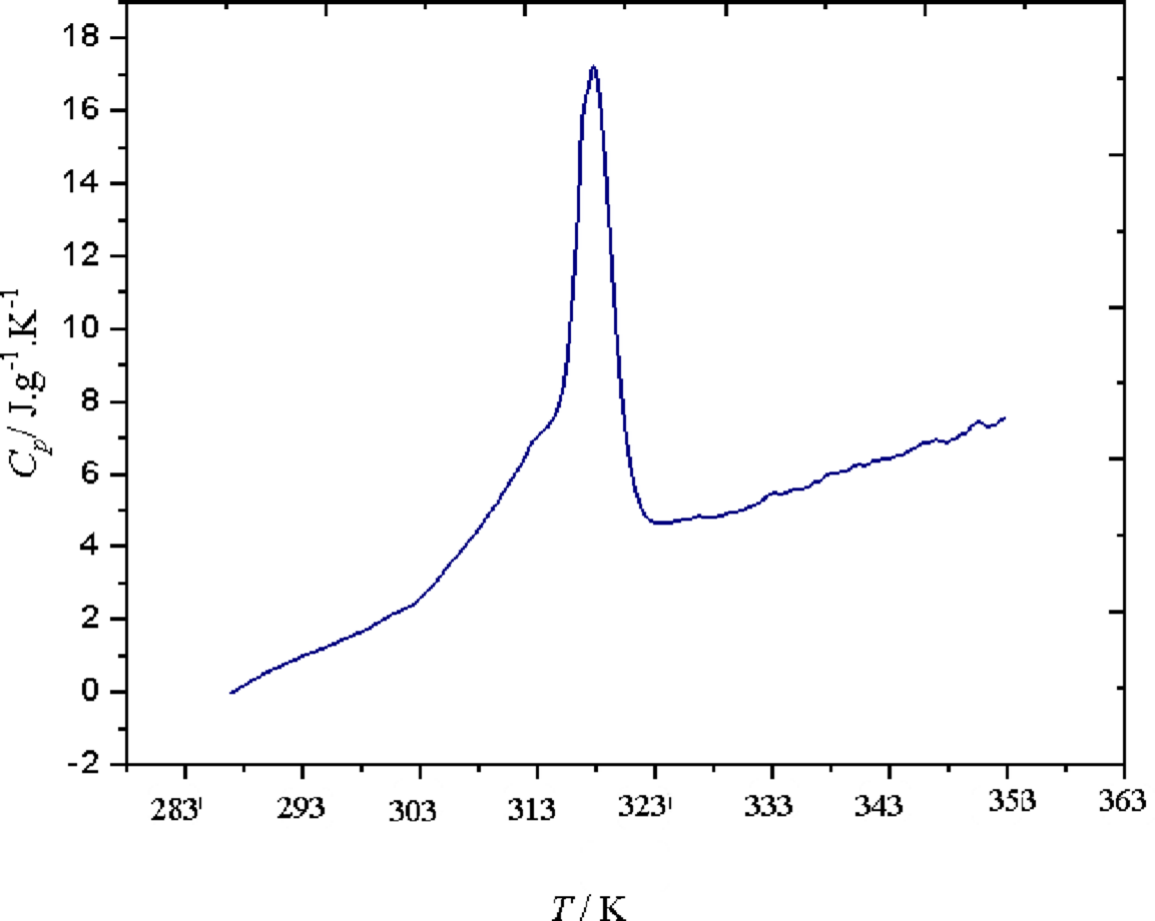
The heat capacity of curcumin.

The phase transition enthalpy (ΔH) of the encapsulated curcumin was measured as 129 J·g⁻¹, which reflects the thermal energy required for melting and utilized to determine the specific heat capacity (C_p_) value (Fig. 5) of the encapsulated drug. The relatively high latent heat value suggests that the PCM shell effectively stores and releases thermal energy, reinforcing its potential in temperature-responsive drug delivery.

The DSC results confirm that curcumin is well integrated within the stearic acid-lauric acid shell, as no significant crystallization peaks of free curcumin are observed. The presence of a single melting transition (PCM-dominated) rather than separate curcumin melting peaks demonstrates homogeneous dispersion within the encapsulation matrix. Compared to free curcumin, which undergoes rapid degradation at elevated temperatures, the encapsulated system provides improved thermal stability and controlled phase transition, beneficial for sustained drug release applications.

### Drug release study

**Fig. 6:**
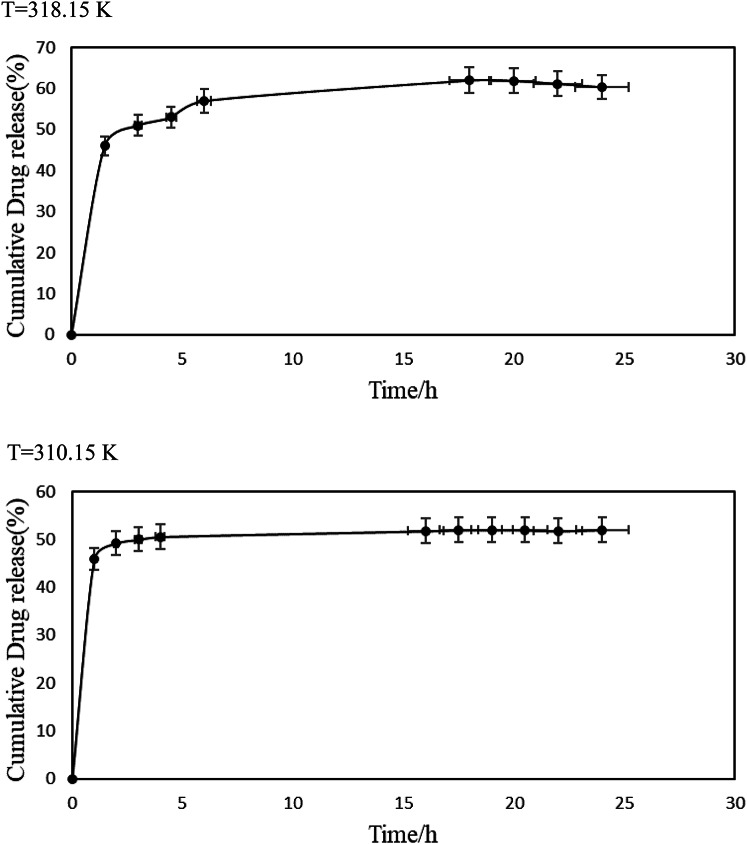
The release kinetics of curcumin demonstrates ~ 50% and about 60% release of curcumin from the curcumin-encapsulated within 24 h at 310.15 K and 318.15 K, respectively, when dispersed in phosphate buffer at physiological pH (7.4).

The release behavior of microencapsulated curcumin was evaluated in phosphate-buffered saline (PBS, pH 7.4) at two physiological temperatures, 310.15 K (37 °C) and 318.15 K (45 °C), over a 24-hour period. The obtained release profile (Fig. 6) provides insights into the controlled release mechanism and temperature dependence of the encapsulated curcumin.

The results indicate a gradual and sustained release of curcumin from the PCM shell over time. After 24 h, approximately 50% of curcumin was released at 310.15 K, while a slightly higher release of ~ 60% was observed at 318.15 K. The controlled release profile suggests that the fatty acid-based shell acts as a diffusion barrier, regulating the release rate and preventing burst effects commonly observed in unencapsulated drugs.

A noticeable increase in drug release was observed at 318.15 K, indicating that the melting of the PCM shell plays a key role in the release process. At 310.15 K, the PCM remains largely in a solid or semi-solid state, resulting in slower diffusion of curcumin. As the temperature approaches or exceeds the PCM melting point (317.91 K), the phase transition enhances drug diffusion, leading to an accelerated release rate at 318.15 K.

The release kinetics of microencapsulated curcumin were also quantitatively analyzed using the Korsmeyer-Peppas model:

2 $$\frac{{{M_t}}}{{{M_\infty }}}=k.{t^n}$$

where $\frac{{{M_t}}}{{{M_\infty }}}$ is the fractional drug release, *k* is the rate constant, and *n* is the release exponent. Log-transformed fractional release data ($\ln \frac{{{M_t}}}{{{M_\infty }}}$) were plotted against $\ln t$ and parameters *n* (slope) and *k* (anti-log of intercept) were derived via linear regression. The calculated parameters are listed in Table 4. This Table shows that the intermediate *n* values (0.5 < *n* < 1.0) indicate anomalous (non-Fickian) transport, where release is governed by both diffusion through the PCM matrix and polymer chain relaxation. The increase in *n* and *k* at 318.15 K reflects the PCM’s phase transition (melting point: 317.91 K), which enhances molecular mobility and accelerates release. This dual mechanism aligns with the system’s designed thermo-responsive behavior, where the solid-to-liquid transition of the PCM shell modulates curcumin diffusion.

**Table 4:** Korsmeyer-Peppas model parameters for Curcumin release from Microcapsules.

Temperature (K)	*n* (Release exponent)	K(h^− *n*^) Rate Constant	*R* ^2^	Release Mechanism
310.15	0.62	0.10	0.985	Anomalous (Non Fickian)
318.15	0.68	0.15	0.992	Anomalous (Non Fickian)

The release kinetic confirms that the stearic acid-lauric acid eutectic mixture provides a temperature-sensitive, sustained-release platform for curcumin. The encapsulation effectively prevents rapid drug loss while allowing controlled diffusion based on physiological temperature changes, making it a promising system for thermal-responsive drug delivery applications.

## Conclusion

In this study, curcumin was successfully microencapsulated within a bio-based phase change material (PCM) composed of stearic acid and lauric acid in a 1:3 molar ratio. The characterization of the synthesized microcapsules was confirmed using FT-IR, SEM, TGA, and DSC analyses. FT-IR spectroscopy confirmed the formation of the PCM shell around curcumin, with the disappearance of free curcumin peaks and the presence of characteristic PCM functional groups. SEM analysis demonstrated the formation of smooth, spherical microcapsules with a uniform size distribution, indicating effective encapsulation. TGA results revealed that the encapsulated curcumin exhibited 98.3% thermal stability up to 423.15 K, significantly improving its stability compared to free curcumin. The DSC analysis demonstrated a single melting peak at 317.91 K with a latent heat of fusion of 129 J·g⁻¹, confirming successful integration of curcumin into the PCM matrix. The drug release study demonstrated a sustained and temperature-responsive release profile, with 50% and ~ 60% of curcumin released at 310.15 K and 318.15 K, respectively, over 24 h. The increased release at higher temperatures highlights the phase transition-driven release mechanism of the PCM, ensuring controlled and prolonged drug delivery.

Overall, the findings confirm that microencapsulation using a stearic acid-lauric acid eutectic mixture enhances the stability, thermal properties, and controlled release of curcumin. This system holds significant potential for temperature-responsive drug delivery applications, offering improved bioavailability and prolonged therapeutic effects. Future work could explore the optimization of PCM compositions to further tailor the release kinetics for specific medical applications.

## Data Availability

All data generated or analysed during this study are included in this article.

## Abbreviations

CUR: Curcumin
PCM: Phase Change Material
SA: Stearic Acid
LA: Lauric Acid
FT-IR: Fourier Transform Infrared Spectroscopy
SEM: Scanning Electron Microscopy
DSC: Differential Scanning Calorimetry
TGA: Thermogravimetric Analysis
PBS: Phosphate Buffered Saline
PVA: Polyvinyl Alcohol
PVP: Polyvinylpyrrolidone
SLS: Sodium Lauryl Sulfate
Cp: Specific Heat Capacity
